# The histamine H3 receptor antagonist thioperamide rescues circadian rhythm and memory function in experimental parkinsonism

**DOI:** 10.1038/tp.2017.58

**Published:** 2017-04-11

**Authors:** D Masini, C Lopes-Aguiar, A Bonito-Oliva, D Papadia, R Andersson, A Fisahn, G Fisone

**Affiliations:** 1 Department of Neuroscience, Karolinska Institutet, Stockholm, Sweden; 2 Department of Physiology and Biophysics, Federal University of Minas Gerais, Belo Horizonte, Brazil; 3 Neuronal Oscillations Laboratory, Division for Neurogeriatrics, Center for Alzheimer Research, Department of Neurobiology, Care Sciences and Society, Karolinska Institutet, Stockholm, Sweden

## Abstract

Parkinson's disease (PD) is a common neurodegenerative disorder, characterized by motor impairment and a wide range of non-motor symptoms, including sleep disorders and cognitive and affective deficits. In this study, we used a mouse model of PD based on 6-hydroxydopamine (6-OHDA) to examine the effect of thioperamide, a histamine H3 receptor antagonist, on circadian activity, recognition memory and anxiety. A partial, bilateral 6-OHDA lesion of the striatum reduces motor activity during the active phase of the 24 h cycle. In addition, the lesion disrupts the endogenous circadian rhythm observed when mice are maintained in constant darkness. Administration of thioperamide to 6-OHDA-lesion mice rescues the normal rest/activity cycle. Moreover, thioperamide counteracts the deficit of novel object recognition produced by 6-OHDA. Our experiments show that this memory impairment is accompanied by disrupted gamma oscillations in the hippocampus, which are also rescued by thioperamide. In contrast, we do not observe any modification of the anxiogenic effect of 6-OHDA in response to administration of thioperamide. Our results indicate that thioperamide may act as a multifunctional drug, able to counteract disruptions of circadian rhythm and cognitive deficits associated with PD.

## Introduction

Nonmotor symptoms are an increasingly urgent problem for the therapy of Parkinson's disease (PD), a common neurodegenerative disorder typically characterized by tremor, rigidity and bradykinesia. Sleep disturbances and circadian rhythm dysfunction affect the vast majority of parkinsonian patients and are often manifested during the early phase of the disease.^1, 2, 3^ Psychiatric conditions, including affective and cognitive disorders, are also commonly associated with PD.^4, 5, 6^ These symptoms are not well characterized and their pharmacotherapy is less developed, prompting the need for the identification of more effective treatments.^5, 6^

Histamine receptors are expressed throughout the brain, where they participate in the regulation of basic homeostatic processes and multiple brain functions, including wakefulness, attention and cognition.^7^ Among the four histamine receptors, the H3 receptor (H3R) has received particular attention as a target for the treatment of neurodegenerative diseases and sleep disorders.^8, 9, 10, 11, 12^ H3Rs are located presynaptically, where they reduce histamine synthesis and release.^13, 14^ Because of their high constitutive activity, H3 autoreceptors are antagonized by inverse agonists,^15^ which promote histamine transmission by acting on the nerve terminals of tuberomammillary neurons.^16, 17^ Administration of H3R antagonists has been shown to improve wakefulness in animal models of narcolepsy, and in narcoleptic patients.^12, 18, 19, 20^ Moreover, these drugs have been shown to enhance cognitive performance and revert the memory loss caused by pharmacological blockade of cholinergic transmission in animal models.^19, 21, 22, 23^

The effects of H3R inverse agonists on wakefulness and cognition are accompanied by enhanced cortical beta (10–30 Hz) and gamma (30–80 Hz) oscillations.^12^ In line with this observation, it has also been shown that activation of H3Rs counteracts gamma oscillations induced by kainate in the hippocampus.^24^ Gamma oscillations have been associated with several higher brain functions, including memory encoding and recall,^25, 26, 27^ facilitation of synaptic plasticity,^28^ and maintenance of selective attention.^29^ Recent studies indicate that dysfunctional plasticity in different regions of the hippocampus is implicated in learning and memory deficits observed in experimental models of PD.^30, 31^ Altogether, these observations raise the possibility that cognitive symptoms in PD may be associated with abnormal oscillatory activity in the hippocampus and suggest the potential utility of pharmacological interventions targeting H3Rs.

In this study, we used a mouse model of PD to examine thioperamide, a selective H3R antagonist,^32^ for its ability to revert dysfunctional circadian rhythm, long-term recognition memory and anxiety-like behavior. Given the involvement of the histaminergic system in the modulation of cognition-relevant oscillatory activity, we also examined the effect of H3R antagonism on hippocampal gamma oscillations.

## Materials and methods

### Animals

Experiments were performed in male C57BL/6N mice (3 months old; Taconic, Tornbjerg, Denmark) and were carried out in accordance with the guidelines of Research Ethics Committee of Karolinska Institutet and Swedish Animal Welfare Agency. All efforts were made to minimize animal suffering and to reduce the number of animals used.

### Drugs

6-Hydroxydopamine (6-OHDA, Sigma-Aldrich, Sweden, Stockholm, Sweden) was dissolved in 0.02% ascorbic acid in saline at a concentration of 4 μg μl^−1^. Thioperamide maleate (Abcam, Cambridge, UK) was dissolved in saline (NaCl 0.9%) and injected at dose of 20 mg kg^−1^.^23, 33, 34^ The control animals were injected with vehicle. Thioperamide and vehicle were administered intraperitoneally in a final volume of 0.01 ml g^−1^ body weight. Repeated administration of thioperamide (for up to 5 consecutive days) did not produce any observable modification in general behavior. Basic chemicals for electrophysiological studies were supplied by Sigma-Aldrich, Sweden and kainic acid by Tocris (Bristol, UK).

### 6-OHDA lesion

Mice were anesthetized with 4% isofluorane and positioned in a stereotaxic frame (David Kopf Instruments, Tujunga, CA, USA) equipped with a heating pad to maintain normothermia. All the animals were injected subcutaneously with 0.1 mg kg^−1^ of Temgesic before surgery. Dopamine depletion was induced by injecting each striatum with 1 μl of 6-OHDA according to the following coordinates (mm): anteroposterior +0.6; mediolateral ±2.2 and dorsoventral −3.2.^35^ The control mice received a sham lesion, consisting of bilateral injections of 1 μl of vehicle. After surgery, the animals were allowed to recover for 2 weeks. The choice of the striatal lesion as a PD model was based on its ability to reproduce a variety of nonmotor symptoms in the mouse.^30, 36^ Moreover, the larger size of the striatum in comparison with other components of the nigrostriatal system (e.g., medial forebrain bundle and substantia nigra pars compacta) allows for a more precise control and better reproducibility of the partial lesion. Following lesion with 6-OHDA, the mice were randomly assigned to saline- or thioperamide-treated groups.

### Assessment of behavioral rhythms

**Figure 1:**
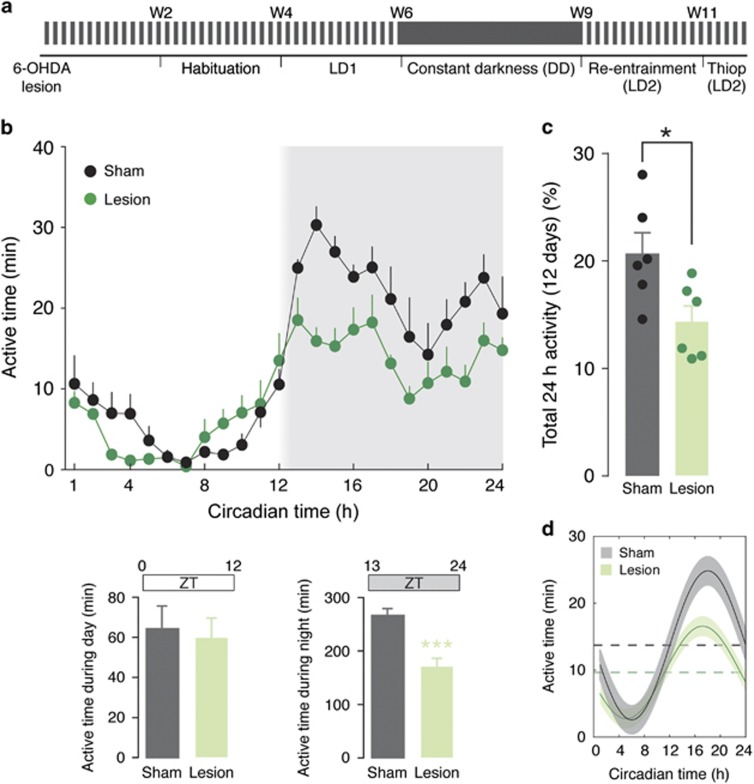
Effect of 6-OHDA lesion on circadian rhythmicity in home cage environment. (**a**) Schematic diagram showing the experimental design. The mice were injected with 6-OHDA and after recovery they were habituated for 2 weeks to the activity monitoring cages. At the end of the habituation period, the effect of the 6-OHDA-lesion was determined under 12 h:12 h light/dark cycle (LD1), followed by release in constant darkness (DD) to determine endogenous circadian rhythm (cf. Figure 2). The mice were then re-entrained (LD2) before testing thioperamide (cf. Figure 3). (**b**) Average activity for sham- and 6-OHDA-lesion mice measured during 12 days (LD1). Shading indicates darkness in reference to ZT time. Lower bar graphs show active time during day (ZT0–ZT12) and night (ZT13–ZT24) in sham- and 6-OHDA-lesion mice. Note the reduction of activity in lesion mice during the night cycle. Data are expressed as means±s.e.m. (*n*=6); ***P<0.001, two-tailed Mann–Whitney *U*-test. (**c**) Bar graph showing active time during LD1. Data represent percentage of active time over total time and are expressed as means±s.e.m. Note the reduction of active time in the 6-OHDA-lesion group; *P<0.05, two-tailed Mann–Whitney *U*-test. (**d**) Waveform cosine curves with 24 h periods fitted to estimate circadian rhythm pattern. Note that the 95% confidence intervals (shadowed areas) of the curves corresponding to sham- (black) and 6-OHDA-lesion (green) mice do not overlap with the respective midline estimating statistic of rhythm (MESOR; broken lines), indicating that the lesion with 6-OHDA does not affect the 24 h rhythmic pattern. 6-OHDA, 6-hydroxydopamine.

Circadian rhythm was assessed using the HM2 rodent activity monitor system (MBROSE, Denmark). This setup consists of two cages (Techniplast polycarbonate cages; 42 × 25 × 15 cm) equipped with two corridors, through which the animals gain access to water and food. The corridors contain a radiofrequency identification antenna to detect individually transponderized mice. The radiofrequency identification detection allows to measure and record real-time activity of multiple rodents in an undisturbed home cage environment. Twelve mice (6 with a sham lesion and 6 with a 6-OHDA lesion) were implanted with subcutaneous radiofrequency identification chips (Microchip T-IS, 13,3 × 2,12 mm, sterile, DataMars, Bedano, Switzerland; at the end of the surgical procedure) and monitored for correct chip placement and surgery recovery. After 2 weeks, the animals were moved into the experimental room and housed (six mice per cage) in the HM2 apparatus. The experiment was performed in a sound-attenuated darkroom, illuminated with a red dim light (positioned 5 m from the cages). During the light phase, the cages were illuminated at 250 Lux. The activity monitor apparatus was placed inside an acoustically isolated rack (Transit unit, Tecniplast, Buguggiate, Italy) with controlled temperature and ventilation. The experimental timeline for the rest/activity analysis is shown in Figure 1a. The mice were first maintained under the same 12 h:12 h light/dark (LD) cycle, to which they had been entrained since birth. The time of light ON is denoted by zeitberg (ZT) 0, and the time of lights OFF by ZT12. Transition between light and dark and vice versa occurred progressively during a period of 1 h. After 2 weeks of habituation to the activity monitor system, baseline activity was recorded for 2 weeks (baseline, LD1). The mice were then released into constant darkness (DD) and the activity data were collected for an additional 3 weeks to assess the endogenous circadian rhythm. After being re-entrained to a 12 h:12 h LD cycle (LD2, 2 weeks), mice with a bilateral partial lesion were injected with thioperamide (ZT11) for 5 consecutive days (LD2-Thiop), while sham animals received vehicle. During the experiment, the animals were checked daily and the cage bedding was changed in a pseudo-random schedule with no interferences during the critical phases of the experiment (no cage changes were made during the first 2 weeks of LD1, the first and third week of DD and 3 days before and during the course of LD2-Thiop). This experiment replicated results previously obtained in the laboratory.

Spontaneous motor activity during rest/activity cycles was determined by measuring the time spent by each animal in the drinking or eating corridors, independently of water or food intake. Data corresponding to 1 h intervals were averaged within controls and 6-OHDA-lesion groups, and plotted as vertical bars in a standard format for graphical representation of rhythm.^37^ Power spectral density was estimated using Fast Fourier Transform, and the power between 12 h and 36 h was integrated, averaged for each group and statistically compared using Student's *t*-test (*P*<0.05). The 95% confidence level was calculated using the bootstrap method. Briefly, the data set was shuffled 10^3^ times and the 95th percentile of the sample was used to determine the upper limit for the spectral analysis.^38^ All the analyses mentioned above were performed in blind using custom-made scripts on Matlab 2009b (MathWorks, Natick, MA, USA). As circadian rhythms can be thought of as smooth rhythms with added noise,^37^ a model consisting of cosine curves with known periods (24 h) was fitted by least squares to the data as an estimate of the pattern of the smooth rhythm. An estimate of the central tendency of activity distribution (Midline Estimating Statistic of Rhythm (MESOR)) was calculated as previously described.^37^

### Novel object recognition test

Mice with a sham or 6-OHDA-lesion were first habituated to the experimental cage (40 × 40 cm plexiglas chamber) for three consecutive days (20 min per day). On the familiarization phase (day 4), the control mice treated with saline (*n*=9) and the lesion mice treated with saline (*n*=6) or thioperamide (20 mg kg^−1^, administered 20 min before familiarization; *n*=9) were introduced in the experimental cage containing two identical objects (gray cylinders) positioned in the back left and right corners of the cage (6 cm from the walls). Each animal was placed in the center of the cage facing the wall opposite to the objects and allowed to explore for 15 min. The mice were tested 24 h after the familiarization phase (day 5). Twenty minutes before the test, the animals were injected with saline or thioperamide and one of the objects in the cage was replaced with a novel object (a cube). Each mouse was then left free to explore for 5 min. The experiment was video-recorded and object exploration (time during which the mouse's nose was in contact with the object or directed toward it at a distance ⩽2 cm) was measured with video tracking software (EthoVision XT, Noldus, The Netherlands). Two measures were considered: first the total time (seconds) spent by the animal exploring the two familiar objects during the familiarization, and second the time spent by the animal exploring the novel object, calculated as percentage of total exploration time (for example, novel/(familiar+novel) × 100) during the test. The exploration time during the test was analyzed by two-way analysis of variance (ANOVA) and Fisher's *post hoc* comparison. The difference in the time spent exploring the novel vs the familiar object is considered an index of recognition memory.

### Elevated plus maze test

The elevated plus maze apparatus was composed of four gray plastic arms (50 × 6 cm), arranged as a cross, located 55 cm above the plane of a laboratory bench and illuminated at 180 Lux. Two arms opposite to each other were delimited by lateral walls (closed arms). The closed and open arms defined a central square area (6 × 6 cm). In this test, the propensity of the animal to avoid the open arm is considered an index of anxiety.^39^ The following experimental groups were examined: control mice treated with saline (*n*=9), lesion mice treated with saline (*n*=6) and lesion mice treated with thioperamide (20 mg kg^−1^; *n*=9) administered 20 min before the test. Each mouse was placed at the center of the maze, facing the open arm away from the researcher, and its behavior was video-recorded for 5 min. The time spent by the animal in each of the three compartments (open, close, center) was measured with EthoVision XT. Entry into a compartment was considered when all four paws were within the same compartment. The data were analyzed by one-way ANOVA followed by Fisher's *post hoc* comparisons.

### Light–dark test

This test is based on the innate aversion of rodents to brightly illuminated areas and on the spontaneous exploratory behavior of rodents in response to mild stressors, i.e., novel environment and light. The light–dark test box (45 × 45 cm, ActiMot2, TSE, Bad Homburg, Germany) was divided into two equally sized compartments (light chamber illuminated at 450 Lux and a dark chamber). Both chambers were connected through an opening (5 × 5 cm) in the center of the arena. The animals were placed in the dark chamber facing a closed wall and tracked via an infrared light beam system. Latency to enter the light chamber was measured with a cutoff time of 360 s. The experiment was terminated whenever the animal had visited the bright compartment for more than 10 s. The data were analyzed with Kruskal–Wallis test followed by Dunn's *post hoc* comparison (*n*=12 mice per group).

### Electrophysiological studies

Sham- and 6-OHDA-lesion mice were deeply anesthetized using isoflurane before being killed by decapitation. The brain was dissected out and placed in ice-cold artificial cerebrospinal fluid (ACSF) modified for dissection. This solution contained (in mm); 80 NaCl, 24 NaHCO_3_, 25 glucose, 1.25 NaH_2_PO_4_, 1 ascorbic acid, 3 NaPyruvate, 2.5 KCl, 4 MgCl_2_, 0.5 CaCl_2_, 75 sucrose. Horizontal sections (350 μm thick) of the ventral hippocampi of both hemispheres were prepared with a Leica VT1200S vibratome (Microsystems, Stockholm, Sweden). Immediately after slicing, the sections were transferred to an interface holding chamber containing standard ACSF (in mm): 124 NaCl, 30 NaHCO_3_, 10 glucose, 1.25 NaH_2_PO_4_, 3.5 KCl, 1.5 MgCl_2_, 1.5 CaCl_2_. The chamber was held at 34 °C for at least 20 min after dissection. It was subsequently allowed to cool to room temperature (~22 °C) for a minimum of 40 min.

Recordings were carried out in hippocampal area CA3 with borosilicate glass microelectrodes, pulled to a resistance of 3–5 MΩ. Local field potentials (LFPs) were recorded in an interface-type chamber (perfusion rate 4.5 ml per minute) at 34 °C using microelectrodes filled with ACSF placed in stratum pyramidale. LFP gamma oscillations were elicited by applying kainic acid (100 nm) to the extracellular bath. The oscillations were allowed to stabilize for 20 min before recordings were carried out. The interface chamber recording solution contained (in mm): 124 NaCl, 30 NaHCO_3_, 10 glucose, 1.25 NaH_2_PO_4_, 3.5 KCl, 1.5 MgCl_2_, 1.5 CaCl_2_.

Interface chamber LFP recordings were performed with a four-channel amplifier/signal conditioner M102 amplifier (Electronics lab, Faculty of Mathematics and Natural Sciences, University of Cologne, Cologne, Germany). The signals were sampled at 10 kHz, conditioned using a Hum Bug 50 Hz noise eliminator (LFP signals only; Quest Scientific, North Vancouver, BC, Canada), software low-pass filtered at 1 kHz, digitized and stored using a Digidata 1322 A and Clampex 9.6 software (Molecular Devices, San Jose, CA, USA).

Power spectral density plots (from 60 s long LFP recordings) were calculated in averaged Fourier-segments of 8192 points using Axograph X (Kagi, Berkeley, CA, USA). Oscillation power was calculated by integrating the power spectral density between 20 and 80 Hz. The integration window was extended to 20 Hz since gamma oscillation frequency is linearly dependent on recording temperature,^40, 41^ which in these experiments is for optimal signal strength held about 3 °C lower than average mouse body temperature. Statistical analysis was performed with unpaired Student's *t*-test.

## Results

### Bilateral partial 6-OHDA lesion of the striatum disrupts circadian rhythm

The control and 6-OHDA-lesion mice were monitored from 3.5 to 6 months of age to determine the effects of partial dopamine depletion on rest/activity patterns (Figure 1a). Following a 2-week habituation period, the effect of the 6-OHDA-lesion was examined on entrainment to a 12 h:12 h light/dark cycle (LD1). Mice with a partial dopamine depletion showed reduced spontaneous motor activity in comparison with control (sham-operated) mice (two-way repeated-measure ANOVA showed a significant effect of the 6-OHDA-lesion, F_(1,14)_=16.52, *P*<0.01; Figures 1b and c). This difference in motor activity was manifested during the dark phase, which corresponds to the active time period for mice (Mann–Whitney *U*-test indicated significant difference between control and lesion mice during night (ZT 13–24, *P*<0.001), but not during day (ZT 1–12, *P*=0.93); Figure 1b, lower panels). Finally, the lesion did not affect rhythmicity, as shown by similar sinusoidal waveforms (defined by cosine fit;^37^ Figure 1d).

**Figure 2:**
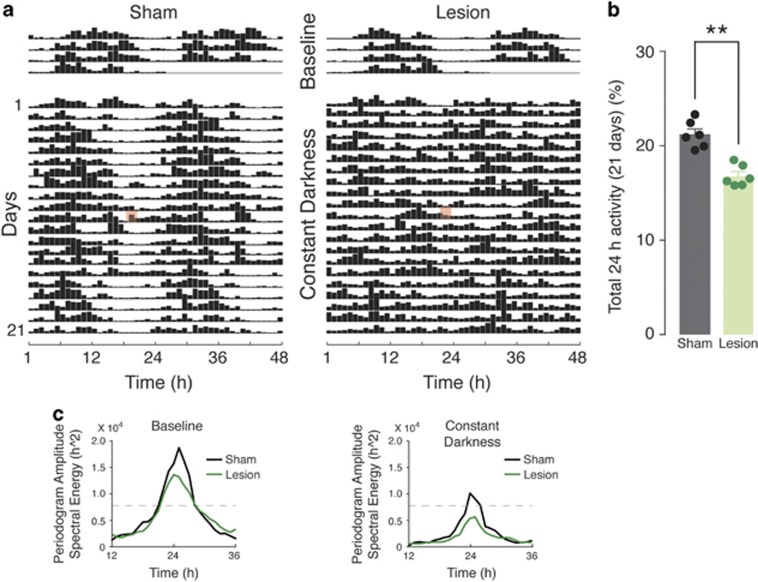
Analysis of endogenous circadian rhythm in 6-OHDA-lesion mice. (**a**) Double-plot actograms showing locomotor activity of control (sham-operated) and 6-OHDA-lesion mice, recorded in LD1 (baseline) and constant darkness (DD). Horizontal lines represent successive days and vertical bars indicate averaged time spent on visiting either of the lateral corridors. Red mark indicates a cage change performed during the course of the experiment. Circadian rhythm is maintained in sham-lesion mice, whereas 6-OHDA-lesion mice display evident arrhythmia. (**b**) Bar graph showing active time during the DD period (21 days). Data represent percentage of active time over total time and are expressed as means±s.e.m. Note that the reduction of active time in the 6-OHDA-lesion group is maintained even in constant darkness; **P<0.01, two-tailed Mann–Whitney test. (**c**) Periodograms obtained by spectral analysis (Fast Fourier Transformation, FFT) confirming persistence and loss of 24 h rhythmicity in sham and lesion mice, respectively. Peaks above the dashed line (indicating the 95% confidence level) were considered significant circadian periods. 6-OHDA, 6-hydroxydopamine; LD, light/dark.

The mice were then released into DD to assess the effect of the 6-OHDA lesion on endogenous circadian rhythmicity. As shown in the double-plot actogram (Figure 2a), control sham-lesion mice maintained well-defined rest/activity periods. In contrast, the activity of 6-OHDA-lesion mice was fragmented throughout the days. Notably, the disruption of endogenous circadian rhythmicity was accompanied by an overall lowered activity level, as observed in LD1 (Figure 2b). In line with these results, power spectral density analysis indicated a 24 h peak amplitude above the 95% confidence interval for control mice, but not for 6-OHDA-lesion mice (Figure 2c). Moreover, the average of the spectral energy centered at 24 h (12 to 36 h) was significantly reduced in the 6-OHDA-lesion mice compared with sham-lesion mice during DD condition (Student's *t*-test, *P*<0.0001) but not during baseline (Student's *t*-test, *P*=0.38; Figure 2c).

### Thioperamide re-establishes normal rest/wake pattern in 6-OHDA-lesion mice

**Figure 3:**
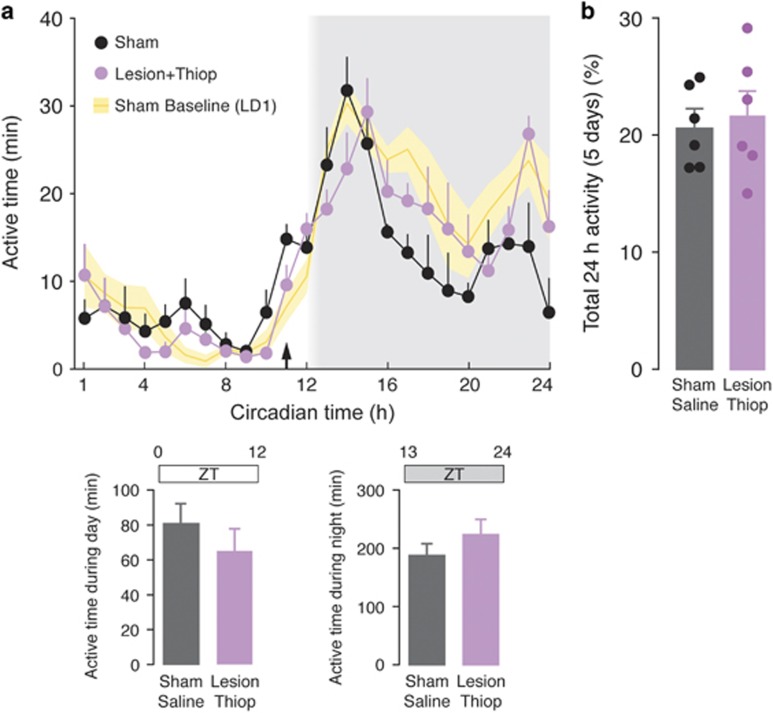
Effect of thioperamide on impaired nighttime activity caused by 6-OHDA. (**a**) Average activity for control (sham) mice and 6-OHDA-lesion mice treated with thioperamide (20 mg kg^−1^) measured during 5 days following re-entrainment from constant darkness (DD) period (cf. Figure 1a). Daily administration of thioperamide 1 h before darkness (ZT11; arrow) re-establishes normal nighttime activity in 6-OHDA-lesion mice. Shading indicates darkness in reference to ZT time (h). Activity in sham mice observed during the LD1 period (cf. Figure 1a) is also shown for comparison (yellow area). Lower bar graphs show active time during day (ZT0–ZT12) and night (ZT13–ZT24) in sham and lesion mice. Note the similar activity in sham and lesion mice treated with thioperamide during the night cycle. Data are expressed as means±s.e.m. (**b**) Bar graph showing active time during the course of the experiment. Data represent percentage of active time over total time and are expressed as means±s.e.m. 6-OHDA, 6-hydroxydopamine; LD, light/dark.

The effect of thioperamide (20 mg kg^−1^) on the rest/activity cycle was then examined in mice with a 6-OHDA-lesion (LD2-Thiop). The mice were first re-entrained for 3 weeks to the 12 h:12 h light/dark cycle (LD2). At the end of this period, sham and lesion mice displayed rest/activity patterns similar to those observed during LD1 (*P*>0.05 for LD1 vs last week of LD2 in both control and 6-OHDA-lesion mice; Mann–Whitney test). Importantly, the difference in rest/activity pattern between control and 6-OHDA-lesion mice was maintained following re-entrainment (*P*<0.05, Mann–Whitney *U*-test). The administration of thioperamide to lesion mice (one daily injection, 1 h before the beginning of the dark phase (ZT11)) re-established levels of activity similar to those observed in control mice treated with vehicle (two-way repeated-measure ANOVA showed no significant difference between sham/vehicle-treated mice and 6-OHDA/thioperamide-treated mice, F_(1,8)_=0.59, *P*=0.47; Figures 3a and b). The effect of thioperamide was exerted during night, which corresponds to the active phase specifically affected by the 6-OHDA lesion (Figure 3a, lower panels).

### Thioperamide rescues 6-OHDA-induced deficit in novel object recognition, but not anxiety-like behavior

**Figure 4:**
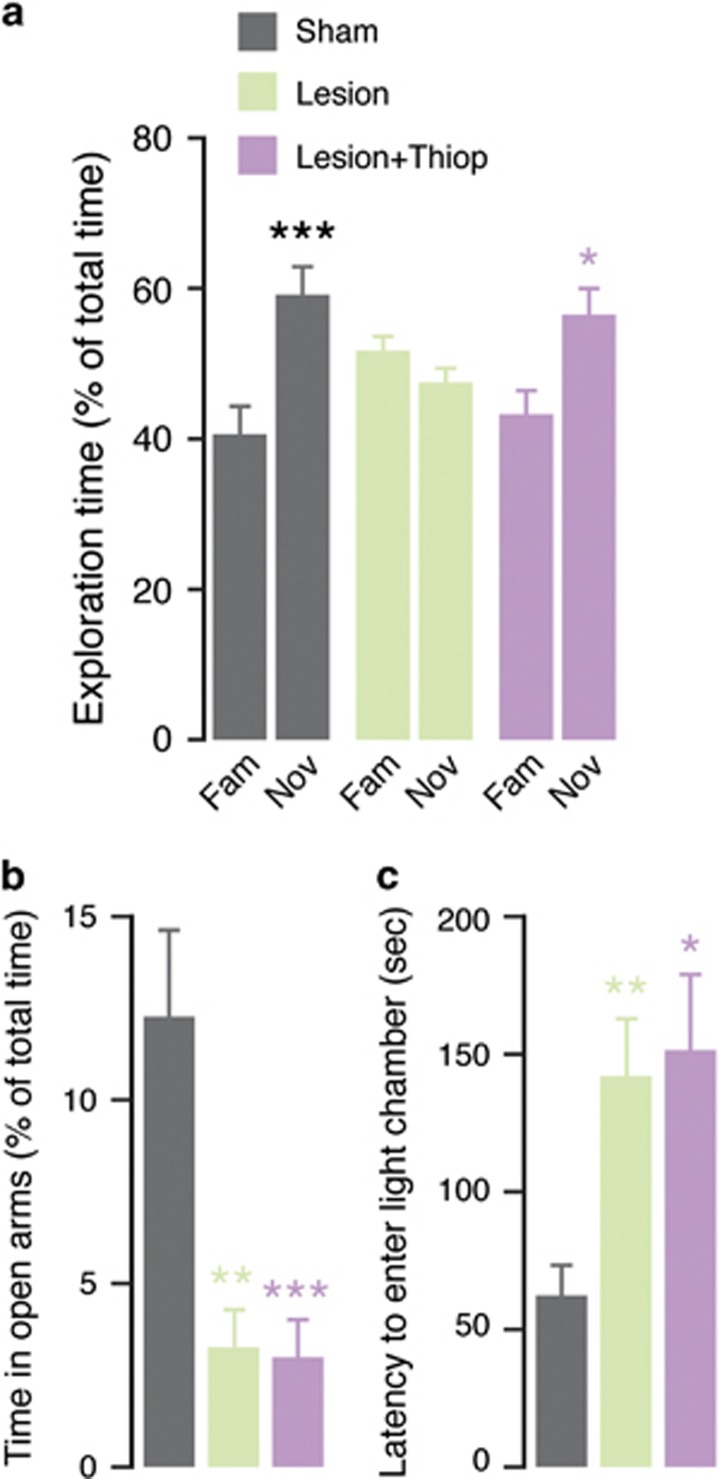
Effect of thioperamide on the novel object recognition deficit and anxiety-like behaviors induced by 6-OHDA. (**a**) Novel object recognition test performed 24 h after familiarization in control (sham) mice, lesion mice and lesion mice treated with thioperamide (20 mg kg^−1^, administered 20 min before familiarization and test). Data indicate the percentage of time spent exploring the familiar or the novel object during a 5 min test, and are calculated as percentage of total exploration time. Bars represent means±s.e.m. *P<0.05 and ***P<0.001 vs novel object same group (two-way analysis of variance (ANOVA) followed by Fisher's *post hoc* comparison). (**b**,**c**) Anxiety-like behavior in control (sham) mice, lesion mice and lesion mice treated with thioperamide, was measured using (**b**) the elevated plus maze test and (**c**) the light–dark box test. (**b**) Histogram showing the percentage of time spent in the open arm of the elevated plus maze during a 5 min-long test. Bars represent means±s.e.m. **P<0.01 and ***P<0.001 vs sham saline (one-way ANOVA followed by Fisher's *post hoc* comparison). (**c**) Histogram showing the latency to enter the light compartment of the light–dark box. Bars represent means±s.e.m. *P<0.05 and **P<0.01 vs sham saline (Kruskal–Wallis followed by Dunn's *post hoc* comparison). 6-OHDA, 6-hydroxydopamine.

The partial lesion with 6-OHDA used in this study impairs long-term novel object recognition.^30^ Therefore, we examined the effect of thioperamide on this cognitive deficit. Sham- and 6-OHDA-lesion mice (from 3.5 to 4.5 months of age) spent similar time exploring the two familiar objects during the familiarization phase (data not shown). In agreement with previous work,^30^ mice with 6-OHDA-lesion failed to recognize a novel object when tested 24 h after the familiarization phase (Figure 4a). This deficit was abolished by thioperamide (20 mg kg^−1^), administered 20 min before familiarization and test phase (two-way ANOVA indicates a significant interaction treatment vs object exploration; *P*<0.05, F_(2,42)_=4.17; Figure 4a).

In addition to cognitive impairment, partial striatal lesion with 6-OHDA results in the emergence of anxiety-like behavior.^36^ To determine a possible anxiolytic effect exerted by blockade of H3R, we examined the effect of thioperamide using the elevated plus maze test. Lesion with 6-OHDA decreased the time spent by the mice (3.5 to 4.5 months of age) in the open arms of the maze and increased the time spent in the closed arms. The administration of thioperamide (20 mg kg^−1^; 20 min before the test) did not modify the anxiogenic effects of the lesion (one-way ANOVA indicates a significant interaction group × time spent in open arms, *P*<0.001, F_(2,19)_=10.34, and in closed arms *P*<0.001, F_(2,19)_=10.24; Figure 4b). Similar results were obtained using the light–dark box test. Mice with a 6-OHDA-lesion displayed longer latency to enter the light chamber, in comparison with control mice. The administration of thioperamide (20 mg kg^−1^; 20 min before the test) did not modify the effect of the lesion (Kruskal–Wallis test, *P*<0.001, F_(2,34)_=5.41; Figure 4c).

### Thioperamide reverts the 6-OHDA-induced degradation of hippocampal gamma oscillations

**Figure 5:**
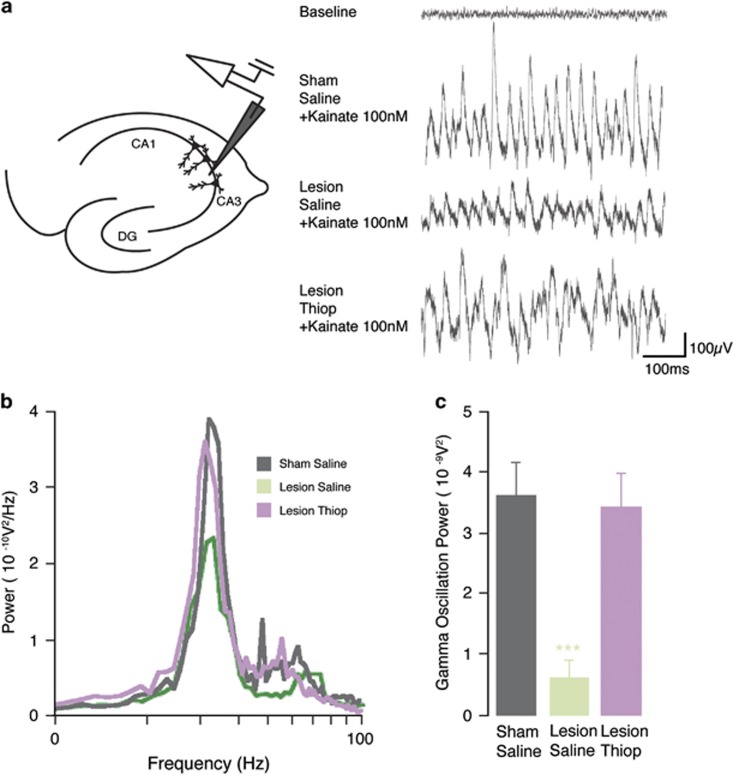
Effect of thioperamide on gamma oscillation deficit in hippocampus induced by 6-OHDA. (**a**) Left panel, diagram showing the positioning of the recording electrode in the CA3 region of the hippocampus. Right panel, example traces of gamma oscillations induced by 100 nm kainate in hippocampal slices from sham mice, and 6-OHDA-lesion mice treated with saline, or with thioperamide (top trace indicates baseline in sham mouse before kainate). (**b**) Representative power spectra of the conditions shown in **a**. (**c**) Summary bar graphs of the conditions shown in **a**. Data are expressed as means±s.e.m. ***P<0.001, Student's *t*-test (unpaired). 6-OHDA, 6-hydroxydopamine.

To test the effect of the 6-OHDA lesion and thioperamide treatment on a cognition-relevant neuronal activity, we recorded the power of kainate-induced gamma oscillations in hippocampal slices (Figure 5a). Gamma oscillations in slices from sham mice injected with saline (3.65±0.57 10^−09^ V^2^, *n*=10, Figure 5, gray) were indistinguishable from gamma oscillations recorded in slices from naive mice (not shown). In contrast, gamma oscillations in slices from 6-OHDA-lesion mice were severely reduced in power (0.65±0.31 10^−09^ V^2^, *n*=9, *P*<0.001, Figure 5, green) indicating that the reduced dopaminergic innervation of the hippocampus caused by the lesion exerts a strong modulatory effect on the network.^42^ Finally, slices obtained from 6-OHDA-lesion mice treated with thioperamide (20mg kg^−1^ administered for three days and killed 20 min after the last injection) generated gamma oscillations indistinguishable from sham mice treated with saline (3.46±0.56 10^−09^ V^2^, *n*=8, *P*=0.83, Figure 5, purple).

## Discussion

In this study, we show that administration of the H3R antagonist thioperamide restores normal circadian rhythmicity in a 6-OHDA mouse model of PD. We also show that thioperamide eliminates the impairment of recognition memory caused by the 6-OHDA lesion. Finally, we found that the memory deficit observed in the mouse model of PD is accompanied by disrupted gamma oscillations in the hippocampus and that this deficit is also corrected by inhibition of H3Rs.

The partial lesion produced by striatal injection of 6-OHDA leads to a reduction of total motor activity during the active (i.e. dark) period of the 24 h cycle, as indicated by the decrease of movement in the activity monitoring cage. Importantly, the lesion also causes a disruption of the endogenous circadian rhythm, as indicated by the severe fragmentation of the activity pattern observed in constant darkness. These results are in line with previous work in the rat, showing that injection of 6-OHDA in the lateral ventricle, which causes a global depletion of dopamine, disrupts circadian wheel-running patterns.^43^ A similar deficit has also been reported in mice overexpressing α-synuclein,^44^ as well as in mice with a loss of striatal dopamine induced by genetic reduction of the vesicular monoamine transporter,^45^ or by disruption of mitochondrial function.^46^

H3Rs are considered a promising target for the development of drugs to treat hypersomnia and H3R antagonists have been shown to promote wakefulness and counteract narcolepsy in animal models.^12^ The observation that, in experimental parkinsonism, thioperamide restores normal motor activity during the active phase of the circadian rhythm indicates the potential efficacy of this drug for the treatment of sleepiness and reduced wakefulness in PD patients.

The present mouse model of PD displays abnormal behaviors indicative of cognitive deficit and mood disorders. In particular, the partial lesion with 6-OHDA results in the loss of long-term recognition memory^30^ and in the development of anxiety-like behaviors.^36^ This offers the advantage of examining the impact of specific therapeutic interventions on a broad range of nonmotor symptoms, including psychiatric symptoms, utilizing the same experimental model.

Reduced alertness, sleepiness and disrupted circadian rhythm are known to affect cognitive function.^47, 48, 49^ This suggests that the ability of thioperamide to re-establish a normal rest/activity pattern in mice with a 6-OHDA lesion may improve their cognitive performance. Our study supports this possibility, indicating that thioperamide rescues long-term recognition memory in the mouse model of PD. This action is most likely mediated by the ability of thioperamide to facilitate brain histamine transmission. In rodents, intracerebroventricular administration of histamine improves performance in the social recognition and inhibitory avoidance memory tests.^50, 51^

In addition to their involvement in the facilitation of histamine transmission, H3R inverse agonists promote the release of other neurotransmitters controlled by H3 heteroreceptors. H3R antagonists exert a particularly pronounced effect on acetylcholine release. Thus, systemic administration of GSK-189254, ABT-239 and pitolisant enhances the extracellular levels of acetylcholine in the cortex (including frontal and cingulate cortex) and hippocampus, which are typically implicated in cognitive functions.^19, 22, 52^ These findings are in line with the ability of H3R antagonists to counteract cognitive deficits produced by scopolamine, a drug that suppresses cholinergic transmission.^19, 22, 52, 53^ Furthermore, blockade of H3R promotes the release of noradrenaline and dopamine, particularly in the mesocortical regions.^19, 22, 52, 53^ However, these effects are less pronounced than those exerted on acetylcholine, suggesting that the ability of thioperamide to reverse the cognitive deficit observed in the mouse PD model may be mediated by positive regulation of the central cholinergic system.

The present study shows that the impairment of long-term recognition memory observed in experimental parkinsonism is associated with degradation of hippocampal gamma oscillations. In the hippocampus, gamma oscillations have been proposed to support memory retrieval by coordinating the activities of the CA3 and CA1 regions.^26^ Gamma frequency activity in the hippocampus has also been correlated with successful encoding of long-term memory,^25^ and increased gamma oscillations are regarded as predictor of memory formation.^27^ These findings suggest that the loss of recognition memory in the mouse PD model may be caused by the parallel disruption of gamma oscillation in the CA3 region of the hippocampus. They also raise the possibility that the restoration of long-term recognition memory produced by thioperamide is at least in part mediated by its ability to rescue gamma oscillations.

The exact mechanism by which thioperamide reverts the deficit in hippocampal gamma oscillation caused by dopamine depletion remains to be elucidated. Thioperamide may re-establish gamma oscillations by blocking inhibitory heteroreceptors and modulating the release of neurotransmitters, including glutamate,^54^ acetylcholine^55^ and catecholamines,^56, 57^ which directly or indirectly impact hippocampal circuits. Activation of H3Rs has been also suggested to inhibit dopamine D1 receptor-mediated responses.^58, 59^ By antagonizing this negative regulation, thioperamide may promote dopamine transmission, thereby counteracting the effect of the partial 6-OHDA lesion.

Augmented histamine transmission, leading to activation of postsynaptic histamine H1 receptors, has been proposed to exert anxiogenic effects.^60, 61^ In contrast with this possibility, Bahi *et al.* reported that administration of an H3R antagonist, which promotes histamine release, counteracts anxiety in naive mice.^62^ In the present study, thioperamide did not affect the anxious phenotype produced by partial dopamine depletion. This highlights the importance of utilizing appropriate animal models of disease when assessing the efficacy of therapeutic interventions for specific pathological conditions.

In conclusion, this study shows that H3R antagonists represent potential multifunctional drugs for the treatment of disturbances of rest/activity cycle and cognitive impairment associated with PD. These results also provide a further validation of a simple toxin-based mouse model for the identification of therapeutic approaches to the treatment of nonmotor symptoms in PD.
